# Myelitis with flaccid paralysis due to Japanese encephalitis: case report and review of the literature

**DOI:** 10.1007/s15010-022-01815-w

**Published:** 2022-04-09

**Authors:** Steven Grewe, Michael Gliem, Daniel B. Abrar, Torsten Feldt, Lars Wojtecki, Victor Tan, Lifea, Shazia Afzal, Sven G. Meuth, Tom Luedde, Hans Martin Orth

**Affiliations:** 1 Department of Gastroenterology, Hepatology and Infectious Diseases, Medical Faculty and University Hospital Düsseldorf, Heinrich-Heine-University, Düsseldorf, Germany; 2 Department of Neurology, Medical Faculty and University Hospital Düsseldorf, Heinrich-Heine-University, Düsseldorf, Germany; 3 Department of Diagnostic and Interventional Radiology, Medical Faculty and University Hospital Düsseldorf, Heinrich-Heine-University, Düsseldorf, Germany; 4 Department of Neurology and Neurorehabilitation, Hospital Zum Heiligen Geist, Kempen, Germany; 5 Department of General Medicine, BIMC Hospital, Bali, Indonesia; 6 Department of Neurology, BIMC Hospital, Bali, Indonesia; 7 Department of Cardiology, Pulmonology and Vascular Medicine, Medical Faculty and University Hospital Düsseldorf, Heinrich-Heine-University, Düsseldorf, Germany; 8 Institute of Clinical Neuroscience and Medical Psychology, Medical Faculty and University Hospital Düsseldorf, Heinrich-Heine-University, Düsseldorf, Germany

**Keywords:** Japanese encephalitis, Myelitis, Flaccid paralysis, Flavivirus, Anterior horn, Indonesia

## Abstract

**Background:**

Japanese encephalitis is an arthropod-borne zoonotic flavivirus infection endemic to tropical and subtropical Asia. A minority of infections leads to a symptomatic course, but affected patients often develop life-threatening encephalitis with severe sequelae.

**Literature review:**

Myelitis with flaccid paralysis is a rare complication of Japanese Encephalitis, which—according to our literature search—was reported in 27 cases, some of which were published as case reports and others as case series. Overall, there is a broad clinical spectrum with typically asymmetric manifestation and partly severe motor sequelae and partly mild courses. Lower limb paralysis appears to be more frequent than upper limb paralysis. An encephalitic component is not apparent in all cases

**Case presentation:**

We herein add the case of a 29 year-old female who developed encephalitis and myelitis with flaccid paralysis during a long-time stay in Indonesia. Diagnostic workup in Indonesia did not clearly reveal an underlying cause. Upon clinical stabilization, the patient was evacuated to her home country Germany, where further diagnostics confirmed Japanese encephalitis virus as the causative agent. The patient has partly recovered, but still suffers from residual paralysis of the upper limb.

**Conclusion:**

Flaccid paralysis is a rare, and likely underdiagnosed complication of Japanese encephalitis, which, to the best of our knowledge, has never been diagnosed outside endemic areas before.

## Introduction

Japanese Encephalitis Virus (JEV) is a zoonotic flavivirus with widespread distribution in tropical Asia. Recurrent epidemics of encephalitis have been recorded in Japan from the 1870s onward, the virus has first been isolated in 1935 [1]. The main vector, *Culex tritaeniorhynchus*, is nocturnal and is mostly found during rainy season in agricultural areas, especially rice fields. Waterfowl and pigs are the main reservoir animals. With an estimated 50.000–175.000 symptomatic human infections per year, Japanese encephalitis is the most frequent viral encephalitis worldwide, children under 14 years are the most affected age group. Symptomatic courses, accounting for less than 1% of infections, usually begin with unspecific flu-like symptoms, but may progress to encephalitis with seizures, psychosis, meningism, coma and various neurological sequelae. Up to one-third of symptomatic cases end fatally [2]. The disease is seldom diagnosed in travelers or expatriates outside Asia [3, 4]. Myelitis with flaccid paralysis is an extremely rarely reported clinical sign, even in endemic areas [5, 6]. Herein, we present a case of an expatriate who contracted Japanese Encephalitis in Indonesia and in the course developed flaccid paresis.

## Case presentation

The previously healthy 29 year-old female had been living in a rural environment with adjacent rice cultivation and livestock farming on Bali, Indonesia for over 2 years. In December 2020, during rainy season, she presented to the emergency department of BIMC hospital with headaches, fever up to 40 °C, and malaise with sudden onset 2 days before. She denied respiratory or gastrointestinal symptoms. Tests for Dengue NS1-Ag and SARS-CoV-2 returned negative, basic blood tests showed no abnormalities. Two days later, she was hospitalized after speaking incoherently and no longer recognizing her family members. On admission, she was somnolent with a Glasgow coma scale (GCS) of 10 (E3V2M5), meningism, a discrete left-sided motor weakness, and a temperature of 37.6 °C. A cerebral Computer Tomography (cCT) scan showed mild cerebral edema. Blood tests were normal apart from an elevated CRP (1.7 mg/dl) and mild leukocytosis (11.330/µl). A lumbar puncture revealed signs compatible with viral meningitis (glucose 72 mg/dl, protein 96.2 mg/dl, cells 7/µl), whereupon acyclovir therapy was initiated. On day five, oxygen desaturation, involuntary movement of the pelvis and hypersalivation were observed and interpreted as seizures related to autoimmune encephalitis, treatment with methylprednisolone (1 g/day) was initiated accordingly. Increasing hypoxemia and hypercapnia led to intubation on day eight. Under mechanical ventilation, arterial blood gas analysis showed adequate parameters, but blood pressure and heart rate were unstable and required vasopressor and antihypertensive treatment interchangeably. Since the patient did not respond to steroid therapy, 27 g of IVIG were administered. On day 11, with sedatives discontinued, the patient was able to open her eyes. Neurological examination revealed slight ptosis on the left eye, hypersalivation and tetraparesis. The autonomous nervous system was still labile with fluctuating blood pressure and heart rate. Besides autoimmune encephalitis, acute demyelinating encephalomyelitis was considered as underlying cause.

**Fig. 1:**
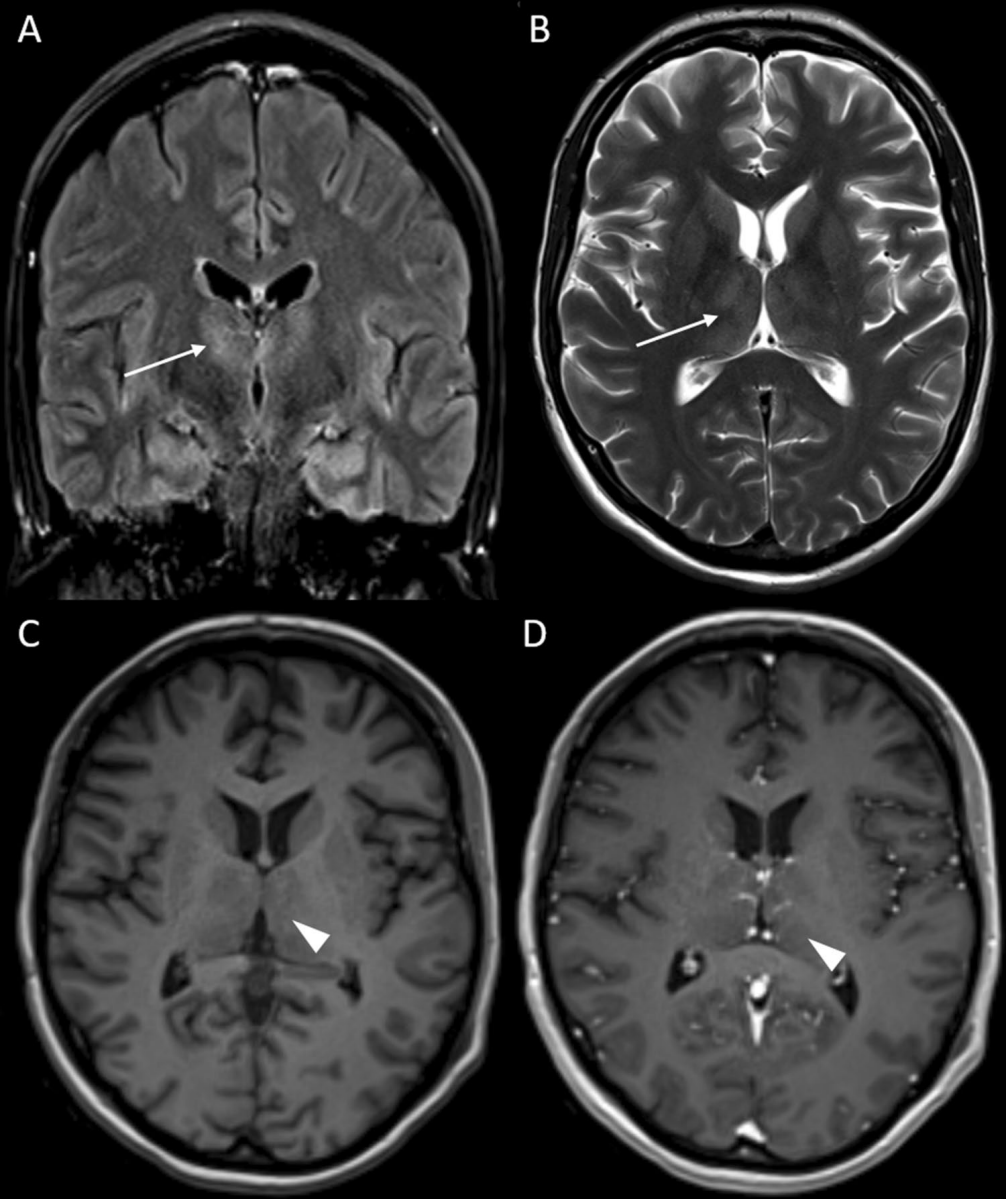
Illustrative 3 T magnetic resonance imaging (MRI) slices of the patient’s brain. A fluid attenuated inversion recovery (FLAIR) sequence in coronal orientation (**A**), a T2-weighted (w) turbo spin echo sequence in transversal orientation (**B**) and a T1w magnetization-prepared rapid gradient echo (MPRAGE) before (**C**) and after intravenous (iv) application of a gadolinium-based contrast agent (GBCA) (**D**) both in transversal orientation are presented. **A** and **B** demonstrate a subtle T2 signal increase (white arrows); and **C** and **D**, a corresponding T1 signal decrease of both thalami (white arrowheads), but no pathological contrast enhancement. The depicted enhancing tubular structures represent blood vessels

**Fig. 2:**
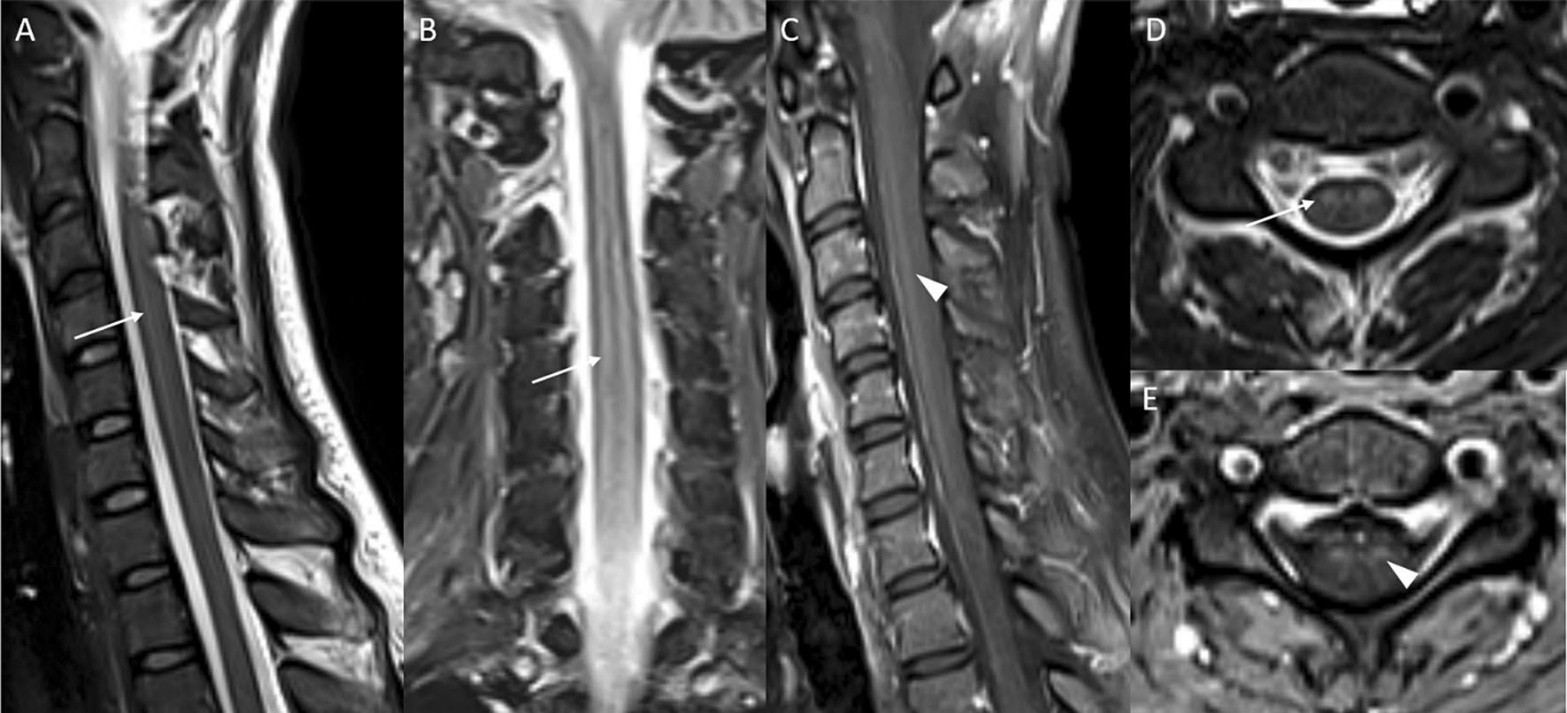
Illustrative 3 T MRI slices of the patient’s cervical spine. C1–Th2 are in sagittal (**A** T2w TSE and **C** T1w TSE with fat-saturation and after iv application of GBCA) and coronal orientation (**B** short tau inversion recovery [STIR]). Representative transversal slices at the C4 level are depicted in **D** (T2w TSE) and E (T1 fs TSA with iv GBCA). Long-range (C2–C6) T2-signal increase of the anterior horn of the myelon (white arrows) with subtle corresponding contrast enhancement (white arrowheads)

Upon further stabilization, she was repatriated on day 18. She was now able to move her right hand (2/5) and both legs (3/5) but unable to elevate her forearms or arms (0/5). Tendon reflexes of the arms were absent but available from both legs. Vital signs were stable, blood testing revealed elevation of CRP (3.2 mg/dl), PCT (0.2 ng/dl), leucocytes, yGT (310 U/l), GPT (73 U/I) and Lipase (209 U/l), all other parameters were within normal range. A follow-up lumbar puncture showed elevated leukocyte counts and a compromised blood–brain barrier (glucose: 80 mg/dl, protein: 75 mg/dl, cells: 41/µl, lactate: 1.9 mmol/l, IgG: 168 mg/l, IgA; 13.2 mg/l, IgM: 40.2 mg/l). IgG and IgM in serum were not elevated (14.6 g/l and 1.59 g/l, respectively) and reibergram indicated intrathecal IgG- and IgM-synthesis. Extensive testing for various neurotropic pathogens yielded negative results for Dengue-, Zika-, Chikungunya-, West Nile-, Nipah-, Mumps-, Measles-, Rubella-, Herpes- and Enterovirus species (including PCR from stool), as well as *Borrelia*, Lues and *HIV*, but showed an elevated IgG (1:1.280; cut-off: < 1:20) and IgM (1:640, cut-off: < 1:20) for JEV in serum and CSF (IgG 1:20, IgM 1:40). The JEV-PCR from CSF, serum and urine returned negative. The patient’s vaccination card showed adequate basic immunizations, but complete lack of travel vaccines (i.e., Hepatitis A, JEV, Rabies, and Typhoid). A follow-up cCT and an EEG showed no abnormalities. Electroneurography showed reduced motor amplitudes of the median nerve, with normal distal latencies and normal sensory amplitudes and velocities corresponding to a pure motoric axonal polyneuropathy or anterior horn cell disorder on the arm without evidence of pathological spontaneous activity or myopathy. A magnetic resonance imaging (MRI) study of the brain demonstrated subtle bilateral thalamic signal abnormalities in terms of T2-hyperintensities and T1-hypointensities without enhancement after intravenous application of a gadolinium-based contrast agent (Fig. 1). MRI of the cervical spine showed long-range distinct T2 signal abnormalities of the anterior horns from C2 to C7 with subtle contrast enhancement (Fig. 2). With continuous need for mechanical ventilation, tracheotomy was conducted on day 23.

Clinical follow-up showed improvement of leg and distal arm motility while proximal arm muscles stayed highly paretic. A follow-up electrophysiological examination on day 32 confirmed a pure motoric axonal neuropathy of arm nerves or anterior horn cell disorder with corresponding fibrillation potentials in the 1st interosseous muscle. The tibial nerve showed normal conduction results. After transfer for neurologic rehabilitation, weaning from tracheostoma was achieved by day 84, but the patient still suffered from hypophonic speech. Fiberendoscopy showed no vocal cord paresis, but lateral pharyngeal paresis, possibly related to affection of the pontine nucleus, as blink-reflex proved pons affection. With neuropsychological tests showing motivation, attention and memory deficits, speech problems could be attributed to the lesions of the lateral, medial and posterior thalamus shown in MRI, with especially bilateral lesions of the (ventro-) lateral and medial thalamus leading to hypophonia [7]. MRI lesions of the spine did not clearly persist in later follow-up; however, electrophysiology still showed signs of anterior spinal damage. One year after symptom onset, our patient showed no more cognitive or psychological impairment, she could move both legs and feet (5/5), and showed improved motility of proximal arms (2/5), arm flection (2/5), extension (3-4/5), finger flection (4/5) and extension (2/5). Tendon reflexes of the arms were absent but clearly present on both legs.

## Literature review

In December, 2021, we conducted a Pubmed query using the search terms “Japanese encephalitis” and (“flaccid paralysis” or “anterior horn” or “myelitis”), which revealed 48 publications. 25 results were not considered because they were not about Japanese Encephalitis and its clinical presentation, the other 23 were screened for further analysis. Five articles were excluded because of redundant data. Eight cases reported no anterior horn involvement, two case series from India mentioned 42 cases of clinically diagnosed anterior horn involvement but lacked specific details, for one case report, only an abstract was available. These publications were also excluded from analysis. The remaining seven publications described a total of 24 cases of Japanese encephalitis with flaccid paralysis and anterior horn involvement. Another series of three cases was found in the references of retrieved articles and included in the analysis.

## Discussion

In our patient, the diagnosis of JE was established based on positive serological findings (IgM and IgG) in CSF and serum, the case therefore fulfills WHO criteria for laboratory-confirmed JE [8]. RT-PCR for JEV from CSF and urine was conducted on day 18 after symptom onset and returned negative. An Indian study reported PCR-positivity in CSF no later than five days from symptom onset [9]. A Chinese study reported no detection of JEV using PCR in urine within 3–9 days from symptom onset, which is in contrast to reliable PCR-detection in urine for other flaviviruses including Dengue, West Nile and Yellow fever [10]. A thorough serological discrimination of infections with other flaviviruses is important, because flavivirus-serology is limited by a high proportion of cross-reactivity [11] and because anterior horn pathologies can also be observed in other infections including Murray Valley encephalitis, tick-borne encephalitis, and West Nile virus [12–14]. Additional serological testing was conducted for other flaviviruses, including Yellow fever virus (YFV), Dengue virus and West Nile virus (WNV), with IgG response for WNV (1:80) and YFV (YFV: 1:640) and negative results for the other viruses. As we later found out, the patient had never stayed in YFV endemic areas, nor had she been vaccinated against yellow fever or any other flavivirus including JEV. Only rarely, JEV-specific IgM is detectable more than 90 days after Infection [15]. The high IgM- and IgG-titers against JEV in contrast to YFV and WNV and the additional detection of antibodies in CSF in combination with the clinical picture, therefore, proves the recent infection with JEV.

CNS damage is caused by direct viral mediated mechanisms (e.g., viral cytolysis) and/or activation of the immune system. Both effects may change over the time course of an infection. It remains, therefore, unclear how and if the initial treatment with glucocorticoids and IVIGs as standard treatment options for suspected autoimmune encephalitis may have influenced the disease [16]. Treatment data of the cases from the literature are scarce and can, therefore, not contribute to the analysis.

As in our patient, thalamic involvement was documented in all encephalitic cases assessed in a Korean study on MRI in JE. In decreasing frequency, hippocampus, midbrain, basal ganglia, neocortex and cerebellum are involved [17]. Data on systematic assessment of spinal involvement are not available. Although anterior horn involvement has been shown in post mortem studies in 1946 [18], the clinical picture of myelitis due to Japanese encephalitis has been described as late as 1991 [19] and corresponding MRI findings in 1997 [20]. Reason for previous underdiagnosing may be false attribution of flaccid paralysis to poliomyelitis, which—until then—used to be the most common cause for this symptom [5]. Since then, only few case reports and case series on the topic have been published. Table 1 gives clinical details on our patient and the 27 cases described in the eight previous publications, which have all been diagnosed in endemic countries in Asia. Including our patient, ten patients were females and 18 males, the patients’ age ranged from 2 to 47 years with only eight patients being 18 years or older.

**Table 1:** Clinical characteristics of the 28 patients with myelitis due to Japanese encephalitis

Author, Year, References	Number of Patients	Age (years)	Sex	Origin of Infection	Clinical Pattern						Recovery				MRI Imaging		
					upper limb	lower limb	Focal muscle wasting only	Tetraplegia	Respiratory failure	Paralysis only	Full	Partial	Poor	Fatal	Spinal MRI conducted	MRI correlates	Images available
Kumar (1991) [19]	3	2, 2, 12	1F, 2 M	India	1	3						3			No		
Misra (1997) [21]	7	2, 12, 14, 15, 18, 28, 47	2F, 5 M	India	6	4	2	2			2	3	2	0	3	1	No
Solomon (1998) [5]	12	3–15, Median 8	3F, 9 M	Vietnam	5	12		5	4	6	1	3	8	0	No		
Chung (2007) [6]	1	22	M	Taiwan	✔	✔			✔			✔			✔	No	No
Narayanan (2017) [22]	1	18	F	India	✔	✔		✔						✔	✔	✔	✔
Ghosh (2017) [23]	1	27	F	India	✔	✔						✔			✔	✔	No
Dev (2020) [24]	1	13	F	India	✔	✔		✔				✔			✔	✔	✔
Shen (2020) [25]	1	30	M	China	✔	✔						✔			No		
Grewe (2022), our patient	1	29	F	Indonesia	✔	✔		✔	✔			✔			✔	✔	✔
Σ	28		10F, 18 M		18	25	2	10	6	6	3	14	10	1	8	5	3

The diagnosis of anterior horn pathology has been established clinically or electrophysiologically in the majority of cases. In our case, electroneurographic and corresponding MRI results confirmed a motor neuron involvement as the correlate of a poliomyelitis-like syndrome, which followed the initial encephalitic stage. In only eight cases, spinal MRI has been conducted (images available in two cases) with five cases showing correlates in the anterior horn. In later MRI follow-up of our patient, anterior horn pathology was not visible anymore despite clinical and electrophysiological proof. The lack of typical MRI signs in three mentioned case reports may be due to imaging at a later stage of the disease. The majority of published cases have occurred more than twenty years back with reduced availability of advanced medical imaging in endemic countries.

While almost all cases (*n* = 25) report lower limb affection, upper limb involvement occurred less frequently (*n* = 18). The extent of paresis in arms and legs was variable: In mild cases (*n* = 2), only focal muscle wasting is documented, while severe cases report quadriplegia (*n* = 10) and respiratory failure (*n* = 6) including our patient. In six cases, flaccid paresis was the only symptom of JE, in one case, flaccid paresis preceded the encephalitic stage of the disease. Generally, asymmetrical distribution is mentioned, which goes in line with the clinical findings in our patient. A partial or poor outcome has been documented in the majority of cases (24/28; 86%), only three patients achieved full recovery (11%).

While JE has usually been considered a childhood disease in endemic areas, data from Korea show a shift in age at diagnosis of JE from childhood to older age [17], which has been confirmed in other areas with unstable JEV transmission and lack of immunity in the population [26]. An analysis of JE cases in travelers has shown a high risk of severe disease in all age groups of non-immune expatriates or travelers to endemic areas [3]. The study may, however, be biased by a different age distribution of travelers in comparison to the general population.

An effective and usually well-tolerated vaccine is available and recommended for long-term travelers to endemic areas [27]. Unfortunately, our patient had moved to Bali without consulting a physician experienced in travel medicine. Her case underlines the necessity of adequate vaccine prophylaxis for all age groups.

## Conclusions

Japanese Encephalitis may cause a broad range of symptoms including rare conditions such as flaccid paralysis. Therefore, Japanese Encephalitis should be considered as a differential diagnosis in travelers to endemic areas presenting with encephalitis and/or myelitis, even in atypical clinical courses. Besides serological diagnostics, MRI may help to guide the treating clinician.

Even though JE remains a rare condition in travelers and expatriates, the course of the disease is often severe or even fatal. Thus, the indication for vaccination should be thoroughly evaluated in all travelers to endemic areas.

## Data Availability

All clinically relevant data have been made available within the manuscript. For data protection reasons, no further clinical data can be made available.

## Abbreviations

CCT: Cerebral computer tomography
CSF: Cerebrospinal fluid
GCS: Glasgow coma scale
HIV: Human immunodeficiency virus
IVIG: Intravenous immunoglobulin
JE: Japanese encephalitis
JEV: Japanese encephalitis virus
MRI: Magnetic resonance imaging
WNV: West Nile virus
YFV: Yellow fever virus
